# Targeting miR-126 in Ph+ acute lymphoblastic leukemia

**DOI:** 10.1038/s41375-023-01933-w

**Published:** 2023-06-09

**Authors:** Junjing Qiao, Dandan Zhao, Le Xuan Truong Nguyen, Fang Chen, Chen Liang, Katrina Estrella, Lucy Y. Ghoda, Nora Heisterkamp, Emanuela C. Marcucci, Ya-Huei Kuo, Guido Marcucci, Bin Zhang

**Affiliations:** 1 Department of Hematological Malignancies Translational Science, City of Hope National Medical Center and Beckman Research Institute, Duarte, CA, USA.; 2 Gehr Family Center for Leukemia Research, City of Hope National Medical Center and Beckman Research Institute, Duarte, CA, USA.; 3 Phase I Clinical Research Center, The Affiliated Cancer Hospital of Zhengzhou University & Henan Cancer Hospital, Zhengzhou, Henan, PR China.; 4 State Key Laboratory of Experimental Hematology, National Clinical Research Center for Blood Diseases, Institute of Hematology & Blood Diseases Hospital, Chinese Academy of Medical Sciences & Peking Union Medical College, Tianjin, PR China.; 5 Department of Systems Biology, City of Hope Beckman Research Institute, Duarte, CA, USA.; 6 These authors contributed equally: Junjing Qiao, Dandan Zhao, Le Xuan Truong Nguyen.

## TO THE EDITOR:

Philadelphia chromosome–positive (Ph^+^) acute lymphoblastic leukemia (ALL) accounts for approximately 25% to 30% of cases of B-cell ALL and is characterized by t(9;22) that created a *BCR::ABL1* fusion gene encoding a chimeric, leukemogenic tyrosine kinase [1]. Historically, patients with this subtype of ALL had a poor prognosis, but implementation of allogeneic hematopoietic cell transplant and, more recently, tyrosine kinase inhibitors (TKIs), and/or blinatumomab (Blincyto, Amgen), a CD3-CD19 bispecific T cell–engaging antibody, early in the treatment has favorably impacted outcome [2, 3]. Nevertheless, dissecting the leukemogenic mechanisms of Ph^+^ ALL may reveal additional “druggable” targets and further improve the outlook of these patients with safer and more effective treatment approaches.

MicroRNAs (miRNAs) are short non-coding RNA molecules that downregulate target messenger (m)RNAs and, in turn, their encoded proteins. MiR-126–3p (miR-126) is highly expressed in normal hematopoietic stem and progenitor cells (HSPC) and maintains self-renewal capacity [4]. Aberrantly increased miR-126 levels have been shown to expand quiescent leukemia stem cells (LSCs) both in acute myeloid leukemia (AML) [5–7] and chronic myeloid leukemia (CML) [8, 9], and initiate and maintain acute lymphoblastic leukemia (ALL) [10]. Of note, while miR-126 supports LSC homeostasis, its production may be blocked by the same aberrant kinases (e.g., FLT3-ITD, BCR::ABL1) that drive leukemic growth [9, 11]. Under these circumstances, LSCs depend on a miR-126 supply from bone marrow (BM) endothelial cells (ECs) [9, 12]. Conversely, while TKIs kill proliferating leukemic blasts, they may restore endogenous production of mature miR-126, which favors persistence and expansion of LSCs, thereby, representing an intrinsic mechanism of cell resistance to these agents [9, 12]. In agreement with this, we observed lower miR-126 levels in BM blasts from BCR::ABL1 ALL mice compared to BM cells from normal wild-type (wt) mice (Supplementary Fig. 1A) and showed that treatment with Dasatinib, a broadly used TKI for Ph+ ALL, increased the endogenous miR-126 (Supplementary Fig. 1B).

**Fig. 1:**
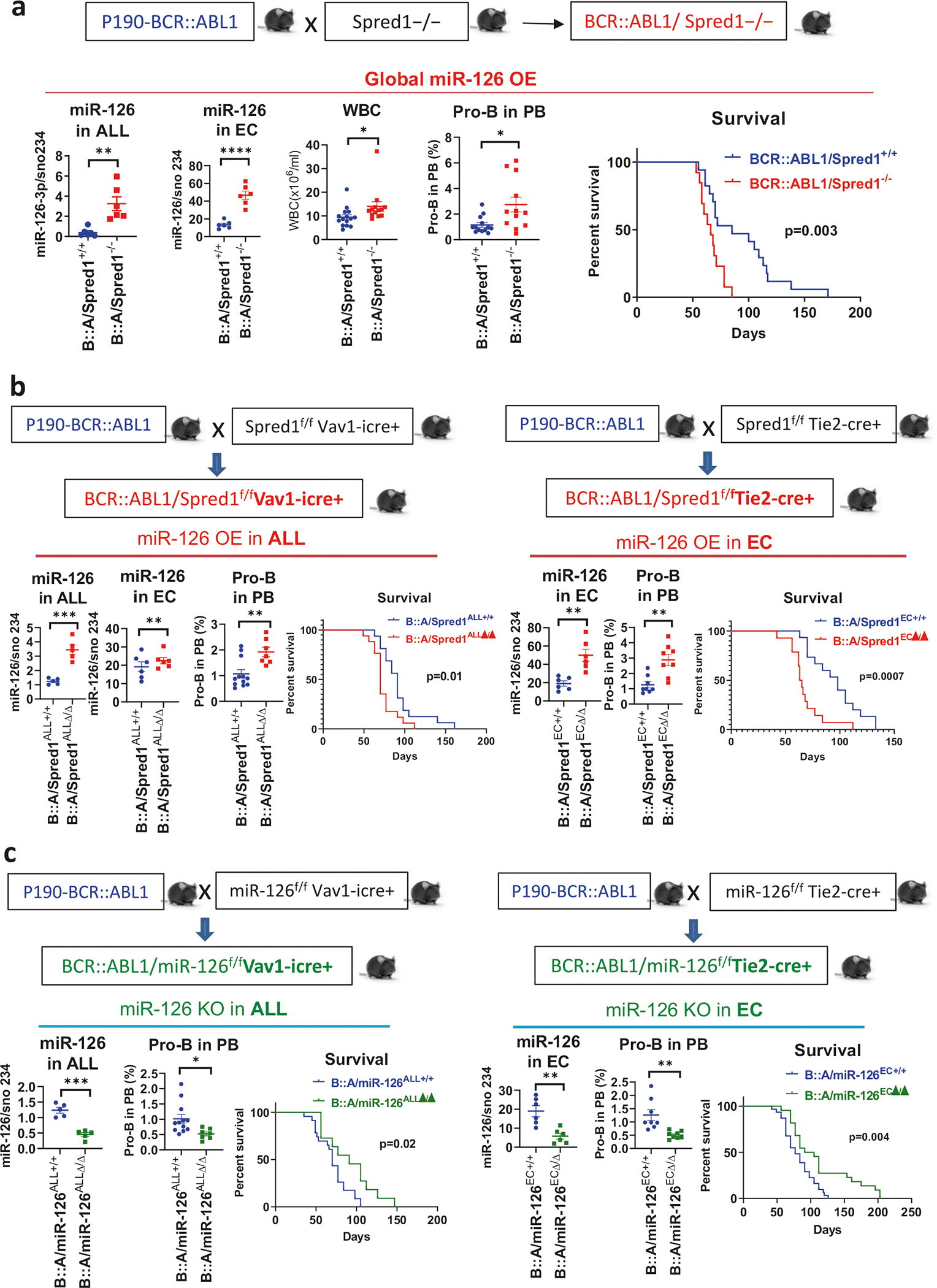
Both hematopoietic and endothelial miR-126 OE promote ALL progression. **a** Schematic design of the mouse crossing and phenotypic comparison. P190-BCR::ABL1 ALL mice were crossed with Spred1 KO (Spred1^−/−^) mice to generate BCR::ABL1/Spred1^−/−^ (miR-126 OE globally) mice. MiR-126–3p levels in BM ALL cells (B220^+^CD19^+^) and endothelial cells (ECs, CD45^−^Ter119^−^CD31^+^) by Q-RT-PCR (*n* = 6 mice per group), white blood cell (WBC) counts (*n* = 12 mice per group), percentage of pro-B blasts (B220^+^CD19^+^CD43^+^IgM^-^) in peripheral blood (PB, *n* = 12 mice per group) by flow cytometry analysis, and survival (*n* = 13 BCR::ABL1/Spred1^−/−^ mice and *n* = 17 BCR::ABL1/Spred1^+/+^ mice) in 6-week-old BCR::ABL1/Spred1^−/−^ versus BCR::ABL1/Spred1^+/+^ mice. **b** Schematic design of the mouse crossing and phenotypic comparison. Left panels: P190-BCR::ABL1 mice were crossed with Spred1^f/f^Vav-icre+ mice to obtain BCR::ABL1/Spred1^f/f^Vav-icre+ (also called BCR::ABL1/Spred1^ALLΔ/Δ^, miR-126 OE in ALL cells) mice. MiR-126–3p levels in BM ALL cells (B220^+^CD19^+^, *n* = 5 mice per group) and ECs (CD45^−^Ter119^−^CD31^+^, *n* = 6 mice per group) by Q-RT-PCR, PB pro-B blasts by flow cytometry analysis (*n* = 12 BCR::ABL1/Spred1^ALL+/+^ mice and *n* = 7 BCR::ABL1/Spred1^ALLΔ/Δ^ mice), and survival (*n* = 16 mice per group) in 6-week-old BCR::ABL1/Spred1^ALLΔ/Δ^ versus BCR::ABL1/Spred1^ALL+/+^ mice. Right panels: P190-BCR::ABL1 mice were crossed with Spred1^f/f^Tie2-cre+ mice to obtain BCR::ABL1/Spred1^f/f^Tie2-cre+ (also called BCR::ABL1/Spred1^ECΔ/Δ^, miR-126 OE in ECs) mice. MiR-126–3p levels in BM ECs by Q-RT-PCR (*n* = 6 mice per group), PB pro-B blasts by flow cytometry analysis (*n* = 8 mice per group), and survival (*n* = 14 mice per group) in 6-week-old BCR::ABL1/Spred1^ECΔ/Δ^ versus BCR::ABL1/Spred1^EC+/+^ mice. c Schematic design of the mouse crossing and phenotypic comparison. Left panels: P190-BCR::ABL1 mice were crossed with miR-126^f/f^Vav-icre+ mice to obtain BCR::ABL1/miR-126^f/f^Vav-icre+ (also called BCR::ABL1/miR-126^ALLΔ/Δ^, miR-126 KO in ALL cells) mice. MiR-126–3p levels in BM ALL (B220^+^CD19^+^) cells by Q-RT-PCR (*n* = 5 mice per group), PB pro-B blasts by flow cytometry analysis (*n* = 12 BCR::ABL1/miR-126^ALL+/+^ mice and *n* = 7 BCR::ABL1/miR-126^ALLΔ/Δ^ mice), and survival (*n* = 23 BCR::ABL1/miR-126^ALL+/+^ mice and *n* = 11 BCR::ABL1/miR-126^ALLΔ/Δ^ mice) in 6-week-old BCR::ABL1/miR-126^ALLΔ/Δ^ versus BCR::ABL1/miR-126^ALL+/+^ mice. Right panels: P190-BCR::ABL1 mice were crossed with miR-126^f/f^Tie2-cre+ mice to obtain BCR::ABL1/miR-126^f/f^Tie2-cre+ (also called BCR::ABL1/miR-126^ECΔ/Δ^, miR-126 KO in ECs) mice. MiR-126–3p levels in BM ECs (CD45^−^Ter119^−^CD31^+^) by Q-RT-PCR (*n* = 6 mice per group), PB pro-B blasts by flow cytometry analysis (*n* = 8 mice per group), and survival (*n* = 22 mice per group) in 6-week-old BCR::ABL1/miR-126^ECΔ/Δ^ versus BCR::ABL1/miR-126^EC+/+^ mice. ALL acute lymphoblastic leukemia, OE overexpression, KO knockout, BM bone marrow, EC endothelial cells, B::A BCR::ABL1, PB peripheral blood, WBC white blood cell. Results shown represent mean ± SEM. Significance values: *p < 0.05; **p < 0.01; ***p < 0.001; ****p < 0.0001.

To fully elucidate the leukemogenic role of miR-126 in BCR::ABL1 ALL, we produced a series of genetically engineered mouse models (GEMMs) of p190-BCR::ABL1 ALL with either global or compartmentalized (hematopoietic or endothelial) miR-126 overexpression (OE) or knockout (KO). The p190-BCR::ABL1 transgenic mice develop ALL, a disease transplantable in congenic recipients [13, 14], and have a median survival of 80 days. To produce BCR::ABL1 ALL mice with global miR-126 OE, we crossed a BCR::ABL1 ALL mouse with a Spred1 KO (Spred1^−/−^) mouse [8] (Fig. 1a). Spred1, an inhibitor of RAS small GTPases, is both a miR-126 target and a negative regulator of miR-126 biogenesis [9]. Spred1^−/−^ mice do not develop leukemia as part of their phenotype, but constitutively express higher levels of miR-126, thereby, representing a functional model of miR-126 OE [8, 9]. Consistent with these results, we observed significantly reduced levels of Cdkn2aip, a reportedly downregulated miR-126 target in miR-126 OE induced B-ALL [10], in BCR::ABL1/Spred1^−/−^ versus BCR::ABL1/Spred1^+/+^ mice (Supplementary Fig. 1C). The BCR::ABL1/Spred1^−/−^ mouse developed a more aggressive ALL with higher white blood cell (WBC) counts and circulating pro-B blasts (B220^+^CD19^+^CD43^+^IgM^−^) and shorter survival (median: 61 vs 80 days, *p* = 0.0006) than the BCR::ABL1/Spred1^+/+^ controls (Fig. 1a; Supplementary Fig. 1D).

To compartmentalize miR-126 upregulation, we then generated BCR::ABL1 ALL GEMMs overexpressing miR-126 in hematopoietic or endothelial cells. We crossed Spred1^flox(f)/f^ mice [8] with Vav-icre+ (Jax lab, #8610) or Tie2-Cre+ (Jax lab, #8863) mice, respectively, and obtained Spred1^f/f^Vav-icre+ (miR-126 OE in hematopoietic cells) and Spred1^f/f^Tie2-Cre+ (miR-126 OE in ECs) mice. We then crossed these mice with p190-BCR::ABL1 mice and, respectively, obtained BCR::ABL1/Spred1^f/f^Vav-icre+ (hereafter called BCR::ABL1/Spred1^ALLΔ/Δ^) and BCR::ABL1/Spred1^f/f^Tie2-Cre+ (hereafter called BCR::ABL1/Spred1^ECΔ/Δ^) mice (Fig. 1b). The BCR::ABL1/Spred1^ALLΔ/Δ^ mouse overexpressed miR-126 in ALL cells, but not in ECs (Fig. 1b, left), had reduced mRNA and protein expression of Cdkn2aip in the ALL cells (Supplementary Fig. 2A) and a more aggressive disease, i.e., higher percentages of circulating pro-B blasts and shorter survival (median: 70 vs 91 days, *p* = 0.01) (Fig. 1b, left) than the BCR::ABL1/miR-126^ALL+/+^ controls. The BCR::ABL1/Spred1^ECΔ/Δ^ mice, which overexpressed miR-126 in ECs, also had a more aggressive ALL than BCR::ABL1/Spred1^EC+/+^ controls, with increased pro-B blasts and shorter survival (median: 64 vs 98 days, *p* = 0.0007) (Fig. 1b, right). Of note, consistent with EC-miR-126 OE, the BCR::ABL1/Spred1^ECΔ/Δ^ mouse presented with an increase in BM CD31^+^Sca-1^high^ ECs and arterioles (Supplementary Fig. 3) that reportedly are a major source of miR-126 for LSCs via extracellular vesicles [9, 12]. In agreement with these results, we observed increased miR-126 and reduced Cdkn2aip levels in BM ALL cells from BCR::ABL1/Spred1^ECΔ/Δ^ mice versus those from BCR::ABL1/Spred1^EC+/+^ control mice (Supplementary Fig. 2B). To confirm the leukemogenic role of the EC miR-126, we transplanted BM cells from p190-BCR::ABL1 mice into Spred1^ECΔ/Δ^ or Spred1^EC+/+^ normal (i.e., non-leukemic) recipients (Supplementary Fig. 4). Spred1^ECΔ/Δ^ recipients developed a more aggressive ALL, with significantly higher WBC counts and pro-B blasts at 4 weeks after transplantation and had a shorter survival (median: 29 vs 38 days, *p* = 0.005) than the Spred1^EC+/+^ recipient controls (Supplementary Fig. 4).

To confirm the relevance of miR-126 to BCR::ABL1 ALL, we also produced BCR::ABL1 ALL GEMMs with miR-126 KO. Firstly, we generated p190-BCR::ABL1 mice with hematopoietic miR-126 KO, by crossing the miR-126^f/f^ mouse with the Vav-icre+ mouse and in turn the miR-126^f/f^Vav-icre+ (miR-126 KO in hematopoietic cells) mouse with the p190-BCR::ABL1 mouse (Fig. 1c, left). We obtained a BCR::ABL1/miR-126^f/f^Vav-icre+ (hereafter called BCR::ABL1/miR-126^ALLΔ/Δ^) mouse with lower miR-126 and higher Cdkn2aip levels in the ALL blasts, lower WBC counts and pro-B blasts, and longer survival (median: 91 vs 70 days, *p* = 0.02) than the BCR::ABL1/miR-126^f/f^/Vav-icre- (BCR::ABL1/miR-126^ALL+/+^) control (Fig. 1c, left; Supplementary Fig. 5A). To compartmentalize the miR-126 KO to ECs, we then crossed the miR-126^f/f^ mouse with the Tie2-cre+ mouse and, in turn, the miR-126^f/f^/Tie2-cre+ (miR-126^ECΔ/Δ^) mouse with the p190-BCR::ABL1 mouse (Fig. 1c, right). The BCR::ABL1/miR-126^f/f^/Tie2-cre+ (also called BCR::ABL1/miR-126^ECΔ/Δ^) mouse had significantly lower EC-miR-126 levels and lived longer (median: 98 vs 77 days, *p* = 0.004; Fig. 1c, right) than the BCR::ABL1/miR-126^EC+/+^ mice. ALL blasts from BCR::ABL1/miR-126^ECΔ/Δ^ mice also had significantly lower miR-126 and higher Cdkn2aip levels than those from BCR::ABL1/miR-126^EC+/+^ mice (Supplementary Fig. 5B). To confirm the leukemogenic role of the EC-miR-126 supply, we also transplanted BM cells from diseased p190-BCR::ABL1 mice into miR-126^ECΔ/Δ^ or miR-126^EC+/+^ normal recipients (Supplementary Fig. 6). MiR-126^ECΔ/Δ^ recipients developed a less aggressive ALL, with significantly lower WBC counts and pro-B blasts, and longer survival (median: 57 vs 42 days, *p* = 0.006) than the miR-126^EC+/+^ recipient controls (Supplementary Fig. 6).

**Fig. 2:**
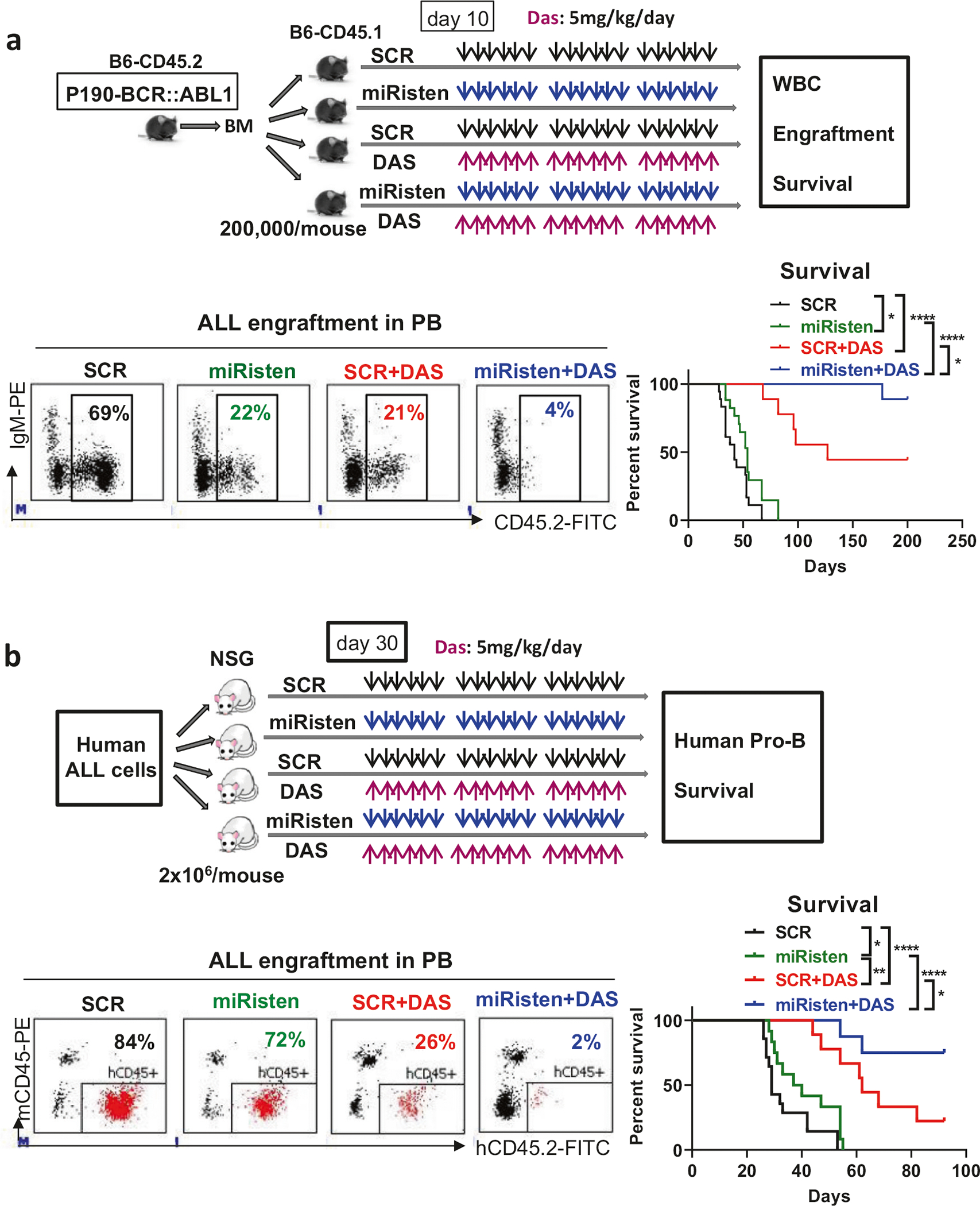
miR-126 downregulation by miRisten in combination with TKI eradiated mouse and human ALL cells in vivo. **a** Schematic design and results of the experiments. BM cells from CD45.2 p190-BCR::ABL1 ALL mice were transplanted into irradiated (2 Gy, X-RAD 320 irradiator) congenic CD45.1 recipients (2 × 10^5^/mouse) by tail vein injection. At day 10 after transplantation, the transplanted mice were divided randomly into 4 groups and treated with SCR (20 mg/kg, IV), miRisten (20 mg/kg, IV), SCR + Dasatinib (5 mg/kg, daily by oral gavage), or miRisten + Dasatinib for 3 weeks. Representative plots of donor ALL cell engraftment (CD45.2+) in PB analyzed by flow cytometry and survival of the treated mice (SCR and miRisten groups: *n* = 18 mice per group; SCR + Dasatinib and miRisten + Dasatinib groups: *n* = 9 mice per group) are shown. b Schematic design and results of the experiments. ALL cells from a Ph+ ALL patient were transplanted into irradiated (1.6 Gy, X-RAD 320 irradiator) NSG mice (2 × 10^6^/mouse) by tail vein injection. At day 30 after transplantation, the mice were randomly divided into 4 groups and treated with SCR (20 mg/kg, IV), miRisten (20 mg/kg, IV), SCR + Dasatinib (5 mg/kg, daily by oral gavage), or miRisten + Dasatinib for 3 weeks. Upon completion of treatment, representative plots of human (h) ALL cell engraftment (hCD45+) in PB and survival of the treated ALL PDX are shown. TKI tyrosine kinase inhibitor, ALL acute lymphoblastic leukemia, WBC white blood cell, PB: peripheral blood, BM bone marrow, Das Dasatinib. Results shown represent mean ± SEM. Significance values: *p < 0.05; **p < 0.01; ****p < 0.0001.

Taken together, these results established a role for miR-126 in sustaining an aggressive p190-BCR::ABL1 ALL phenotype and led us to hypothesize miR-126 as a potentially druggable target. We previously reported on miRisten, a novel anti-miR-126 oligonucleotide, that was effectively taken up and downregulated miR-126 in ECs and leukemic cells [9, 12] (see also Supplementary Fig. 7). To test the activity of miRisten against BCR::ABL1 ALL blasts in vivo, we synchronized a cohort of mice for ALL development by transplanting CD45.2 p190-BCR::ABL1 ALL blasts into congenic CD45.1 recipients. The transplanted mice, divided randomly into 4 groups, were then treated with SCR (20 mg/kg, IV), miRisten (20 mg/kg, IV), SCR + Dasatinib (5 mg/kg, daily by oral gavage), or miRisten + Dasatinib for 3 weeks (Fig. 2a). Increased expression of Cdkn2aip (Supplementary Fig. 8A) and longer survival (median survival: 54 vs 42 days, *p* = 0.03; Fig. 2a) were observed in miRisten-treated mice compared with SCR-treated controls. Of note, miRisten plus TKI had the best outcome with a significantly increased survival compared with SCR plus TKI (median survival: not reached vs 127 days, *p* = 0.03; Fig. 2a; Supplementary Fig. 8B). Of note, 9 out of 10 mice in the miRisten+TKI-treated group remained alive after 200 days with no evidence of leukemic cells (CD45.2+) at necropsy (Supplementary Fig. 8C), suggesting that they were potentially cured.

To assess the relevance of these results to human disease, we transplanted primary human Ph+ ALL cells into NSG mice. At day 30 after transplantation, the mice were randomly divided into 4 groups and treated with SCR (20 mg/kg, IV), miRisten (20 mg/kg, IV), SCR + Dasatinib (5 mg/kg, daily by oral gavage), or miRisten + Dasatinib for 3 weeks, followed by assessment of human cell engraftment in PB, BM and spleen and survival (Fig. 2b). MiRisten-treated mice had significantly increased levels of the miR-126 target Cdkn2aip (Supplementary Fig. 8D) and a significantly reduced burden of human (h) ALL pro-B blasts (hCD45^+^CD19^+^CD34^+^) in PB, BM and spleen at the end of treatment and lived longer (median: 38.5 vs 29 days, *p* = 0.04; Fig. 2b; Supplementary Fig. 8E) than SCR-treated mice. TKI-treated mice also had a significantly reduced ALL pro-B blasts in PB, BM and spleen upon completion of treatment and lived longer than miRisten-treated and SCR-treated mice (median survival: 62 vs 38.5 vs 29 days for TKI vs miRisten vs SCR; TKI vs miRisten*, p* = 0.001; TKI vs SCR: *p* < 0.0001; Fig. 2b; Supplementary Fig. 8E). Mice treated with miRisten plus TKI had the lowest disease burden and lived significantly longer than the other groups (e.g., median survival of miRisten + TKI vs SCR + TKI: not reached vs 62 days, *p* = 0.03; Fig. 2b; Supplementary Fig. 8E). Only 2 out of 8 mice in the miRisten+TKI-treated group vs 7 out of 9 mice in SCR + TKItreated group died after monitoring them for 100 days. At this time point, those surviving mice had no evidence of human cells (hCD45^+^) in PB, BM or spleen, suggesting that they were potentially cured.

In summary, our results support a leukemogenic role of miR-126 in BCR::ABL1 ALL cells. Of note, while we used Spred1 KO to induce endogenous miR-126 upregulation and obtain functional miR-126 OE models of BCR::ABL1 ALL, loss of Spred1, a negative regulator of the pro-leukemogenic RAS-MAPK signaling [8, 15], might itself contribute to the leukemic phenotype, independently of miR-126 levels. Nevertheless, our current and previous work both support Spred1 KO models as useful tools to study molecular mechanisms and pharmacological targeting of miR-126 OE-dependent leukemogenesis [10]. Accordingly, we showed that the miR-126 inhibitor miRisten, alone or in combination with TKI, had significant antileukemic activity in these models. To this end, we showed 90% complete remission and leukemia-free survival of p190-BCR::ABL1 ALL mice and 75% of Ph+ ALL patient-derived xenografts (PDXs) treated with miRisten and TKI. With recent emerging data that support chemotherapy-free approaches for Ph + ALL [2, 3], miR-126 targeting may provide an additional therapeutic opportunity for these otherwise poor-risk patients.

## Supplementary Material

Supplementary Information

## Data Availability

Requests for original data may be submitted via e-mail to the corresponding author (bzhang@coh.org).
