# Supplementary material for: Targeting miR-126 in Ph+ acute lymphoblastic leukemia
Source: Leukemia. Author manuscript; Available in PMC 2024 Jul 1. (PMC10317835; doi:10.1038/s41375-023-01933-w)
Supplement: Supplementary Information [file NIHMS1909808-supplement-Supplementary_Information.docx]

**Supplementary Information**

**Supplementary Materials and Methods**

**Supplementary Figure 1**

**Supplementary Figure 2**

**Supplementary Figure 3**

**Supplementary Figure 4**

**Supplementary Figure 5**

**Supplementary Figure 6**

**Supplementary Figure 7**

**Supplementary Figure 8**

**Materials and Methods**

**Human samples**

ALL samples were obtained from patients who have not received prior TKI treatment from the Hematopoietic Tissue Biorepository (HTB) core of City of Hope National Medical Center (COHNMC). ALL samples used in this study are P190-BCR/ABL positive confirmed by FISH analysis and QPCR. Mononuclear cells were isolated using Ficoll separation.

ALL specimens were collected from patients registered at COHNMC. ALL patients and healthy donor participants at COHNMC were consented to Institutional Review Board (IRB) approved biorepository protocols, COH IRB 18067 or 06229, respectively, in accordance with an assurance filed with and approved by the US Department of Health and Human Services and met all requirements of the Declaration of Helsinki.

### Animal studies

Spred1*^−/−^* and Spred1^f/f^ mice (from Dr. Akihiko Yoshimura, Keio University School of Medicine, Japan) were crossed with Vav-iCre+ (Jax lab, #8610) or Tie2-Cre+ (Jax lab, #8863) and then with p190-BCR/ABL (from Dr. Nora Heisterkamp, City of Hope) mice to obtain the following strains: BCR/ABL/Spred1*^−/−^*, BCR/ABL/Spred1^f/f^/Vav-iCre, and BCR/ABL/Spred1^f/f^/Tie2-Cre (all CD45.2 B6 background). MiR-126^f/f^ mice (from Dr. Calvin J Kuo, Stanford University School of Medicine) were crossed with Vav-iCre or Tie2-Cre and then with p190-BCR/ABL mice to obtain BCR/ABL/miR-126^f/f^/Vav-iCre and BCR/ABL/miR-126^f/f^/Tie2-Cre strains (all CD45.2 B6 background). Recipient mice in the CD45.1 B6 background (from NCI) were used to allow tracking of donor CD45.2 cells after transplant. Recipient mice were 6 to 8 weeks old and were irradiated at 2 Gy within 24 hours before transplantation. The number of mice for each study group was chosen based on the expected endpoint variation (i.e., engraftment rate and latency period of leukemia) and mice availability from distinct strains. The mice of the same gender and age were randomly divided into groups. Investigators were blinded to mice genotypes while performing treatment or monitoring engraftment or survival. Mouse care and experimental procedures were performed in accordance with federal guidelines and protocols approved by the Institutional Animal Care and Use Committee at COHNMC.

**Flow cytometry analyses**

To determine disease progression in ALL mice, mouse cells were obtained from peripheral blood (PB), BM (from both tibias and femurs) and spleen and strained with the following anti-mouse antibodies: B220 (CD45R) (FITC, clone RA-6B2, cat# 14-0452-82, eBioscience), CD19 (APC-eFluor780, clone eBio1D3, cat# 47-1093-82, eBioscience), CD43 (PE, clone eBioR2/60, cat# 12-0431-82, eBioscience), IgM (APC, clone 11/41, cat# 17-5790-82, eBioscience), CD45.1 (PE-Cy7, clone A20, cat# 560578, BD), and CD45.2 (BV421, clone 104, cat# 562895, BD). To determine the engraftment rate of human ALL cells in NSG mice, PB, BM and spleen cells were stained with anti-human CD45 (FITC, clone HI30, cat#304038, BD), CD19 (PE, clone HIB19, cat#555413, BD) and CD34 (APC, clone 4H11, cat#17-0349-42, BD) antibodies. All analyses were performed on a Fortessa x20 flow cytometer (BD Biosciences) and data were analyzed by BD FACSDiva or FlowJo software.

**Gene expression by Q-RT-PCR**

To measure the miRNA expression, total RNA was extracted using the miRNeasy Mini Kit (Qiagen, Valencia, CA). Reverse transcription using MultiScribe™ Reverse Transcriptase and Q-PCR analysis of miR-126 (assay ID: 2228, ThermoFisher) and Cdkn2aip (assay ID: Mm00549617_m1 for mouse and Hs00214157_m1 for human, ThermoFisher) were performed according to the manufacturer’s protocol. SnoRNA234 (assay ID: 1234, ThermoFisher) and β2m (assay ID: Mm00437762_m1 for mouse and Hs00187842_m1 for human; ThermoFisher) were used as an internal control for mouse miRNA. The results are presented as a log2-transformed ratio according to the 2^–ΔCt^ method (ΔCt=Ct of target –Ct of reference).

**Immunoblotting analysis**

Mouse BM cells from various mouse models or from the treated ALL murine and PDX models were washed in ice-cold PBS and subsequently lysed in buffer containing 1 mM phenylmethanesulfonylfluoride and 10 mM protease inhibitor cocktail. 30 μg of each cell lysate was incubated with anti-CDKN2AIP antibody (ab140519, Abcam) and then separated on NuPAGE 4–12% gradient gels (Invitrogen) and immunocomplexes were visualized with enhanced chemiluminescence reagent (Thermo Scientific, Lafayette, CO).

**Immunofluorescent staining and 3D confocal imaging of long bones**

Long bones (tibias or femurs) from mice were processed, sectioned and imaged as described previously^1^ with ad-hoc modifications. Briefly, the bone was dissected from mice and immediately fixed with 4% paraformaldehyde (PFA) for 4 hours. Then the bone was washed with PBS at 4^0^C and processed for decalcification by incubating in decalcification solution (0.5M EDTA, pH 7.4-7.6) for 24 to 48 hours. The bone was washed with PBS and continuously incubated with ice-cold cryoprotectant (CPT) solution at 4^0^C for 24 hours. After incubating with embedding media (EBM) for 45 min at 60^0^C, the bone was embedded in a tissue mold, which was dried at room temperature (RT) for 30 min and stored at -80^0^C at least overnight. Tissue sections were cut at -23^0^C using a microtome with a thickness of 40 µm and then transferred to a microscope slide for staining. The slides were incubated with fluorescent conjugated antibodies (SCA-1-PE, CD31-FITC; 1:250 dilution) overnight at 4^0^C. Then, the slides were washed with PBS and nuclear staining was performed with mounting solution containing 4′,6-diamidino-2-phenylindole (DAPI, BD). 3D imaging was performed using a confocal microscope (Zeiss, LSM880) and analyzed using the Zen program (Zeiss).

***In vivo* treatment of p190-BCR/ABL murine and PDX model**

BM cells (CD45.2) were obtained from diseased p190-BCR/ABL mice and transplanted by tail vein injection (2x10^5^ cells/mouse) into recipient mice (CD45.1) irradiated at 2 Gy (X-RAD 320 irradiator). At day 10 after transplantation, these mice were treated with SCR (20mg/kg, 6 times a week by vein injection), miRisten (20mg/kg, 6 times a week by vein injection), SCR + Dasatinib (5mg/kg, daily by oral gavage), or miRisten + Dasatinib for 21 days. After 3 weeks of treatment, mice were monitored for leukemia burden in PB and survival. To obtain the ALL PDX model, ALL cells from a Ph+ ALL patient were transplanted by tail vein injection into irradiated (1.6 Gy, X-RAD 320 irradiator) NSG mice (2x10^6^ cells/mouse). At day 30 after transplantation, these mice were randomly divided into 4 groups and treated with SCR (20mg/kg, 6 times a week by vein injection), miRisten (20mg/kg, 6 times a week by vein injection), SCR + Dasatinib (5mg/kg, daily by oral gavage), or miRisten + Dasatinib for 21 days. After 3 weeks of treatment, a cohort of mice were euthanized and PB, BM and spleen cells were analyzed for ALL cell burden, and another cohort of mice was followed for survival after discontinuation of treatment.

**Statistical analysis**

Comparison between two groups was examined by two-tailed, unpaired t-test or paired t-test. The log-rank test was used to assess significant differences between survival curves. All statistical analyses were performed using Prism version 8.0 software (GraphPad Software). *In vivo* experiments were performed using 8–22 mice in each group.  Sample sizes chosen are indicated in the individual figure legends and were not based on formal power calculations to detect prespecified effect sizes. P values ≤ 0.05 were considered significant. Results shown represent mean ± SEM. *, p < 0.05; **, p < 0.01; ***, p < 0.001; ****, p < 0.0001; ns, not significant.

1 Kusumbe, A. P., Ramasamy, S. K., Starsichova, A. & Adams, R. H. Sample preparation for high-resolution 3D confocal imaging of mouse skeletal tissue. *Nat Protoc* **10**, 1904-1914, doi:10.1038/nprot.2015.125 (2015).

**Supplementary Figure 1. miR-126 OE promotes ALL progression. A-B** MiR-126-3p levels in BM cells from p190-BCR::ABL1 ALL or normal wild-type mice (**A**) and in BM cells from p190-BCR::ABL1 ALL mice treated *in vitro* with DMSO or Dasatinib (100nM) for 24 and 48 hours (**B**), analyzed by Q-RT-PCR. **C** Expression of Cdkn2aip in BM ALL cells from BCR::ABL1/Spred1^+/+^ and BCR::ABL1/Spred1^−/−^ mice analyzed by Q-RT-PCR and western blot. **D** Representative plots showing the frequency of pro-B blasts (B220^+^CD19^+^CD43^+^IgM^-^) in the PB of BCR::ABL1/Spred1^−/−^ (miR-126 OE) and BCR::ABL1/Spred1^+/+^ mice by flow cytometry analysis. Abbreviation: DMSO: Dimethylsulfoxide; OE: overexpression; ALL: acute lymphoblastic leukemia. B::A: BCR::ABL1. PB: peripheral blood. Results shown represent mean ± SEM. Significance values: *, p<0.05; ***, p<0.001; ****, p<0.0001.

**Supplementary Figure 2. Reduction of miR-126 target Cdkn2aip in miR-126 OE ALL mice**. **A** Expression of Cdkn2aip in BM ALL cells from BCR::ABL1/Spred1^ALL+/+^ and BCR::ABL1/Spred1^ALLΔ/Δ^ (miR-126 OE in ALL) mice analyzed by Q-RT-PCR and western blot. **B** Expression of miR-126 and Cdkn2aip in BM ALL cells from BCR::ABL1/Spred1^EC+/+^ and BCR::ABL1/Spred1^ECΔ/Δ^ (miR-126 OE in ECs) mice analyzed by Q-RT-PCR and western blot. Abbreviation: OE: overexpression; ALL: acute lymphoblastic leukemia. B::A: BCR::ABL1. PB: peripheral blood. Results shown represent mean ± SEM. Significance values: *, p<0.05; **, p<0.01; ***, p<0.001.

**Supplementary Figure 3. BCR::ABL1/Spred1^ECΔ/Δ^ (miR-126 OE in ECs) mice had increased CD31^+^SCA-1^high^ EC-lined arteriole vessels compared to BCR::ABL1/Spred1^EC+/+^ control mice.** CD31 (FITC) and SCA-1 (PE) immunofluorescence (IF) staining of CD31^+^SCA-1^high^ EC-lined vessels (i.e., arterioles, indicated by yellow arrows) in the tibias from BCR::ABL1/Spred1^EC+/+^ and BCR/ABL/Spred1^ECΔ/Δ^ mice. Three mice per group were analyzed with similar results. Scale bar represents a size of 400µm. Abbreviation: ALL: acute lymphoblastic leukemia; OE: overexpression; EC: endothelial cells.

**Supplementary Figure 4. EC miR-126 levels mediate ALL progression.** BM cells from p190-BCR::ABL1 ALL mice were transplanted into irradiated (X-ray, 2Gy) Spred1^ECΔ/Δ^ (EC-miR-126 OE) or Spred1^EC+/+^ normal (i.e., non-leukemic) recipients (2x10^5^/mouse). WBC counts and PB pro-B blasts (B220^+^CD19^+^CD43^+^IgM^-^) measured at 4 weeks after transplantation and survival of Spred1^ECΔ/Δ^ versus Spred1^EC+/+^ recipients are shown. Abbreviation: ALL: acute lymphoblastic leukemia; EC: endothelial cells; BM: bone marrow; OE: overexpression; WBC: white blood cell; PB: peripheral blood. Results shown represent mean ± SEM. Significance values: **, p<0.01; ***, p<0.001.

**Supplementary Figure 5. Increase of miR-126 target Cdkn2aip in miR-126 KO ALL mice**. **A** Expression of Cdkn2aip in BM ALL cells from BCR::ABL1/miR-126^ALL+/+^ and BCR::ABL1/miR-126^ALLΔ/Δ^ (miR-126 KO in ALL) mice analyzed by Q-RT-PCR and western blot. **B** Expression of miR-126 and Cdkn2aip in BM ALL cells from BCR::ABL1/miR-126^EC+/+^ and BCR::ABL1/miR-126^ECΔ/Δ^ (miR-126 KO in ECs) mice analyzed by Q-RT-PCR and western blot. Abbreviation: KO: knockout; ALL: acute lymphoblastic leukemia. BM: bone marrow; B::A: BCR::ABL1. Results shown represent mean ± SEM. Significance values: *, p<0.05; **, p<0.01; ***, p<0.001.

**Supplementary Figure 6. EC miR-126 levels mediate ALL progression.** BM cells from p190-BCR::ABL1 ALL mice were transplanted into miR-126^ECΔ/Δ^ (miR-126 KO in ECs) or miR-126^EC+/+^ normal (i.e., non-leukemic) recipients (2x10^5^/mouse). WBC counts and PB pro-B blasts (B220^+^CD19^+^CD43^+^IgM^-^) measured at 4 weeks after transplantation and survival of miR-126^ECΔ/Δ^ versus miR-126^EC+/+^ recipients are shown. Abbreviation: EC: endothelial cells; ALL: acute lymphoblastic leukemia; BM: bone marrow; KO: knockout; WBC: white blood cell; PB: peripheral blood. Results shown represent mean ± SEM. Significance values: *, p<0.05; **, p<0.01.

**Supplementary Figure 7. Efficient uptake of miRisten in mouse and human ALL cells. A** MiRisten-Cy3 uptake measured by flow cytometry at 4 hours and miR-126-3p levels by Q-RT-PCR at 24 hours after addition of CpG-miR-126 inhibitor (miRisten) conjugated with Cy3 (miRisten-Cy3) in BM ALL cells from p190-BCR::ABL1 ALL mice. Uptake test was replicated twice with similar results. **B** MiRisten-Cy3 uptake measured by flow cytometry at 4 hours and miR-126-3p levels by Q-RT-PCR at 24 hours after addition of miRisten-Cy3 in ALL cells from Ph+ ALL patients (n=3). Abbreviation: ALL: acute lymphoblastic leukemia. Results shown represent mean ± SEM. Significance values: **, p<0.01; ***, p<0.001.

**Supplementary Figure 8.** **MiR-126 downregulation by miRisten in combination with TKI eradiated mouse and human ALL cells *in vivo*. A** Expression levels of miR-126 target Cdkn2aip in BM ALL cells from CD45.1 recipient mice transplanted with BM cells from CD45.2 p190-BCR::ABL1 ALL mice (2x10^5^/mouse) and then treated with SCR (20mg/kg, IV), miRisten (20mg/kg, IV), SCR + Dasatinib (5mg/kg, daily by oral gavage), or miRisten + Dasatinib for 3 weeks, analyzed by Q-RT-PCR and western blot. **B** WBC counts (n=15 mice per group) and PB pro-B (B220^+^CD19^+^CD43^+^IgM^-^) blasts (n=15 mice per group) in the 4 groups of treated mice from **A**. **C** Plots of leukemic cell engraftment (CD45.2+) in the BM of the remaining nine miRisten+TKI treated mice at necropsy, analyzed by flow cytometry. Related to **Figure 2a**. **D** Expression levels of miR-126 target Cdkn2aip in BM ALL cells from the NSG mice transplanted with Ph+ ALL patient cells and then treated with SCR (20mg/kg, IV), miRisten (20mg/kg, IV), SCR + Dasatinib (5mg/kg, daily by oral gavage), or miRisten + Dasatinib for 3 weeks, analyzed by Q-RT-PCR and western blot. **E** Human (h) pro-B (hCD45^+^CD19^+^CD34^+^) blast engraftment in PB, BM and spleen of the treated ALL PDX mice from **D**. Related to **Figure 2b**. Abbreviation: TKI: tyrosine kinase inhibitor; ALL: acute lymphoblastic leukemia; PDX: patient-derived xenograft; DAS: Dasatinib; WBC: white blood cell; PB: peripheral blood. Results shown represent mean ± SEM. Significance values: *, p<0.05; **, p<0.01; ***, p<0.001; ****, p<0.0001.
